# Plasma biomarkers distinguish Boston Criteria 2.0 cerebral amyloid angiopathy from healthy controls

**DOI:** 10.1002/alz.70010

**Published:** 2025-03-29

**Authors:** Ryan T. Muir, Sophie Stukas, Jennifer G. Cooper, Andrew E. Beaudin, Cheryl R. McCreary, Myrlene Gee, Krista Nelles, Nikita Nukala, Janina Valencia, Kristopher M. Kirmess, Sandra E. Black, Michael D. Hill, Richard Camicioli, Cheryl L. Wellington, Eric E. Smith

**Affiliations:** ^1^ Calgary Stroke Program University of Calgary Calgary Alberta Canada; ^2^ Hotchkiss Brain Institute, Departments of Radiology University of Calgary Calgary Alberta Canada; ^3^ Community Health Sciences, Departments of Radiology University of Calgary Calgary Alberta Canada; ^4^ Clinical Neurosciences University of Calgary Calgary Alberta Canada; ^5^ L.C Campbell Cognitive Neurology Research Unit, Dr Sandra Black Centre for Brain Resilience and Recovery, and Hurvitz Brain Sciences Program, Sunnybrook Research Institute University of Toronto North York Ontario Canada; ^6^ Department of Pathology and Laboratory Medicine, Djavad Mowafaghian Centre for Brain Health University of British Columbia Vancouver British Columbia Canada; ^7^ Departments of Radiology University of Calgary Calgary Alberta Canada; ^8^ Division of Neurology, Department of Medicine University of Alberta Edmonton Alberta Canada; ^9^ C2N Diagnostics St. Louis Missouri USA; ^10^ Neuroscience and Mental Health Institute University of Alberta Edmonton Alberta Canada

**Keywords:** Alzheimer's disease, beta‐amyloid, cerebral amyloid angiopathy, glial fibrillary acidic protein, hemorrhagic stroke, neurofilament light chain, phosphorylated tau, plasma biomarkers

## Abstract

**INTRODUCTION:**

Cerebral amyloid angiopathy (CAA) is characterized by the deposition of beta‐amyloid (Aβ) in small vessels leading to hemorrhagic stroke and dementia. This study examined whether plasma Aβ_42/40_, phosphorylated‐tau (p‐tau), neurofilament light chain (NfL), and glial fibrillary acidic protein (GFAP) differ in CAA and their potential to discriminate Boston Criteria 2.0 probable CAA from healthy controls.

**METHODS:**

Plasma Aβ_42/40_, p‐tau‐181, NfL, and GFAP were quantified using single molecule array (Simoa) and Aβ_42/40_ was also independently quantified using immunoprecipitation liquid chromatography mass‐spectrometry (IPMS).

**RESULTS:**

Forty‐five participants with CAA and 47 healthy controls had available plasma. Aβ_42/40_ ratios were significantly lower in CAA than healthy controls. While p‐tau‐181 and NfL were elevated in CAA, GFAP was similar. A combination of Aβ_42/40_ (Simoa), p‐tau‐181, and NfL resulted in an area under the curve of 0.90 (95% confidence interval: 0.80, 0.95).

**DISCUSSION:**

Plasma Aβ_42/40_, p‐tau‐181, and NfL differ in those with CAA and together can discriminate CAA from healthy controls.

**Highlights:**

- Participants with CAA had reduced plasma Aβ_42/40_ ratios compared to controls.
- Plasma p‐tau‐181 and NfL concentrations are elevated in CAA compared to controls.
- Plasma GFAP was similar in CAA and controls.
- Together, plasma Aβ_42/40_, p‐tau‐181, and NfL had excellent discriminability for CAA.

## 1 INTRODUCTION

Spontaneous intracerebral hemorrhage (ICH) confers high morbidity and mortality and may be the harbinger of an underlying neurodegenerative and cerebrovascular disorder: cerebral amyloid angiopathy (CAA).^1^, ^2^, ^3^, ^4^ Accurately diagnosing CAA is important as it identifies those with elevated risk of ICH recurrence and dementia.^5^, ^6^, ^7^, ^8^ Further, in an era of emerging immunotherapies targeting beta‐amyloid in Alzheimer's disease (AD), it is imperative to identify those with CAA given the risk of amyloid related imaging abnormalities (ARIA).^9^, ^10^, ^11^, ^12^, ^13^

CAA is characterized by the progressive deposition of beta‐amyloid within the media and adventitia of cortical and leptomeningeal small vessels.^14^, ^15^ On the other hand, AD is typified by extracellular, intraparenchymal, beta‐amyloid (Aβ) plaques, and intracellular neurofibrillary tangles of hyperphosphorylated tau.^16^ Up to 80% of patients with AD have CAA on pathology.^7^, ^16^

Currently, the Boston Criteria 2.0, which integrate core clinical and magnetic resonance imaging (MRI) criteria, inform a diagnosis of *probable* and *possible* CAA.^4^ However, limitations of these diagnostic criteria include: the requirement of MRI for full sensitivity; hemorrhagic manifestations occur onat the latest stage of CAA; CAA cannot be discerned in mixed states where neuroimaging features of hypertensive arteriopathy coincide with those of CAA; and the criteria may have limited diagnostic discriminability in some settings.^4^, ^15^, ^17^, ^18^

Plasma biomarkers are less invasive, accessible, and scalable evaluations of candidate pathophysiologic substrates of CAA and thus could potentially aid in earlier disease detection. Plasma Aβ (the Aβ_42/40_ ratio) and phosphorylated tau (p‐tau) herald the presence of AD in reference to cerebrospinal fluid (CSF) and positron emission tomography (PET) signatures.^19^, ^20^ Furthermore, neurofilament light chain (NfL), an indicator of axonal injury, is elevated in AD and other neurodegenerative states, and emerging evidence suggests glial fibrillary acidic protein (GFAP), a marker of astrocyte activation, is also elevated in AD.^19^ Plasma biomarkers in CAA are under‐explored.^19^ In CSF, persons with CAA have reduced Aβ_40_ compared to both controls and AD; reduced Aβ_42_ compared to controls (though similar Aβ_42_ compared to AD); reduced Aβ_42/40_ compared to controls (though higher compared to AD); and elevated total‐tau and phosphorylated‐tau compared to controls, but lower than AD.^21^ The current study evaluated (1) whether plasma Aβ_42_, Aβ_40,_ Aβ_42/40_ ratio, p‐tau‐181, GFAP, and NfL differ in CAA compared to controls and (2) biomarker discriminative performance individually and in combination.

## 2 METHODOLOGY

### 2.1 Research ethics

The Functional Assessment of Vascular Reactivity in Small Vessel Disease‐II (FAVR‐II) Study is a prospective cohort study approved by the University of Calgary and University of Alberta Research Ethics Boards (REB). Written informed consent was provided by all participants. The current cross‐sectional analysis includes FAVR‐II baseline data.

**RESEARCH IN CONTEXT**

1. **Systematic review**: A PubMed search identified cohort studies that evaluated cerebrospinal fluid (CSF) or plasma biomarkers of beta‐amyloid (Aβ), total‐tau, p‐tau, and/or neurofilament light chain (NfL) in cerebral amyloid angiopathy (CAA). While studies of CSF suggest that there are reductions in Aβ_40_, Aβ_42_, Aβ_42/40_, and elevations in tau in CAA compared to controls, the few studies that have examined plasma biomarkers in CAA have yielded variable results.
2. **Interpretation**: Using two independent modern methodologies to quantify Aβ (immunoprecipitation liquid chromatography mass‐spectrometry and single molecule array [Simoa]), as well as Simoa to evaluate p‐tau‐181, NfL, and glial fibrillary acidic protein (GFAP), this study identified differences in plasma biomarkers in those with CAA compared to controls. While there was no difference in plasma Aβ_40_ or GFAP, we did observe reductions in the Aβ_42/40_ ratio and elevations in p‐tau‐181 and NfL.
3. **Future directions**: These results invite future work to evaluate plasma biomarkers in larger external validation cohorts in cases with neuropathologically confirmed CAA and with comparisons to cohorts with AD.

### 2.2 Study population

Participants ≥ 55 years of age were recruited between 2015 and 2023 from stroke prevention or cognitive clinics. This age was selected as, at the time of study inception, Boston Criteria version 1.5 was used to inform study inclusion criteria. Healthy controls were community volunteers between the ages of 60–85 years without stroke or cognitive impairment ascertained through medical history and neuropsychological evaluation.^22^ Additionally, participants with substantive alcohol or drug abuse affecting cognition; Montreal Cognitive Assessment (MoCA) scores <13; or brain diseases other than CAA (e.g., leukoencephalopathy, multiple sclerosis, neoplasms, Parkinson's Disease, and other neurologic disorders) were not eligible. Those with CAA presented either with ICH ≥ 90 days before enrollment, transient focal neurologic episodes (TFNEs), or cognitive decline.^22^ Participants with cognitive decline could include cases with possible mixed dementia, but patients meeting clinical criteria for Lewy body disease, frontotemporal dementia, or other non‐AD non‐vascular cognitive disorders were excluded. A diagnosis of probable CAA was ascertained through magnetic resonance imaging (MRI). A 3.0T MRI (Calgary: Discovery MR750, GE Healthcare, Waukesha, USA; Edmonton: Prisma, Siemens Healthineers, Forchheim, Germany) was obtained including T1‐weighted, T2‐weighted, proton‐density‐weighted‐T2, fluid attenuated inversion recovery (FLAIR) and T2*‐weighted gradient recalled echo (T2*GRE) images. An experienced neurologist (E.E.S.) evaluated images for criterion of Boston Criteria 2.0.^4^

MRI scans were visually reviewed, blinded to other participant information, for intracerebral hemorrhages, microbleeds, cortical superficial siderosis (cSS), white matter hyperintensity (WMH), and perivascular spaces (PVS) following definitions of the Standards for Reporting Vascular Changes on Neuroimaging.^23^ A six‐point ordinal CAA small vessel disease score was computed.^24^ WMH were rated according to the Fazekas scale,^25^ and high WMH was defined, as in the CAA small vessel disease score, as either subcortical hyperintensity score of 2 or 3 or a periventricular score of 3.^24^ To apply the Boston criteria 2.0, the presence of the WMH multi‐spot sign (>10 spots) was recorded.^4^ PVS were rated according to the Wardlaw scale, and high centrum semiovale PVS count (CSO‐PVS) was defined as >20 PVS (grade 3 or 4, according to the Wardlaw scale),^26^ which is a supportive criterion for CAA according to the Boston criteria 2.0. cSS was categorized as focal (≤3 affected sulci) versus disseminated (>3 sulci) as in previous studies of CAA.^27^

### 2.3 Plasma collection

At baseline, two 4 mL plastic ethylenediaminetetraacetic acid (EDTA) tubes of blood were collected. The tubes were mixed by gently inverting 8–10 times then centrifuged at 1200 × *g* for 15 min. Subsequently, 1.5 mL of plasma from each tube was aliquoted into 0.5 mL polypropylene cryovials (stored at ‐80°C until analyzed).

### 2.4 Plasma biomarker analysis

Analyses to quantify all plasma biomarker concentrations (pg/mL) were conducted blinded to participant status. Plasma Aβ_42_ and Aβ_40_ concentrations were measured independently by two labs using different methods: (1) immunoprecipitation liquid chromatography mass‐spectrometry (IPMS) analytical platforms previously described and validated at C_2_N Diagnostics (Saint Louis, MO, USA)^28^, ^29^, ^30^, ^31^, ^32^ and (2) single molecule array (Simoa) at the University of British Columbia. Second, we analyzed plasma Aβ_42_ and Aβ_40_ concentrations with Simoa utilizing the Quanterix Ltd Neurology‐4‐Plex‐E (N4PE) Advantage Kit (cat ID 103670, lot 503768), a multiplex assay that quantifies Aβ_40_, Aβ_42_, NfL, and GFAP. Plasma p‐tau‐181 was measured using version 2.1 Advantage Kit (cat ID 104111, lot 503843). Simoa assays were performed using an HD‐X Analyzer following the manufacturer's instructions. All plasma samples were analyzed in one batch and in duplicate with the average of the values used in subsequent analyses. Each assay included an eight‐point calibrator curve, two provided kit controls, and three plasma samples from healthy individuals.

### 2.5 Validation against external normative data

We determined whether the individual plasma biomarker levels for healthy controls and CAA were within or outside of age‐specified normative reference intervals (RIs) for plasma NfL, GFAP, p‐tau‐181, and the ratio of Aβ_42/40_ derived from individuals between the ages of 3 to 79 years in the Canadian Health Measures Survey (hosted by the Statistics Canada Biobank) by Cooper et al.^33^ According to each participant's age, cutoffs were defined as: an upper limit (upper 95% confidence interval [CI] of the 95th percentile) for p‐tau‐181, NfL, and GFAP and a lower limit (lower 95% CI of the 5th percentile) for the Aβ_42_/Aβ_40_ ratio.^33^ These RIs were generated using a different lot of the same Simoa N4PE assay and version 2.0 of the p‐tau‐181 assay. Thus, a conversion factor, published by Quanterix, was used to adjust the p‐tau‐181 values between version 2.0 and 2.1 of the assay. For participants who were 80 years of age and older, we used the published normative cutoffs for 79 years of age.^33^ Odds ratios and 95% CIs evaluate whether those with CAA had a higher, lower or equal odds of biomarker values falling outside of these normative cut offs compared to healthy controls. Additional details are provided in the Data Supplement.

### 2.6 Statistical analyses

Statistical analyses were conducted in Stata v18.0 (StataCorp LLC, Texas) and figures with plasma biomarker values generated using GraphPad Prism (v10.2.0). Continuous variables were evaluated for normality. Continuous variables were summarized as the mean and standard deviation (SD) if normally distributed or median and interquartile range (IQR) if not. Normally distributed variables were compared using the Student's t‐test, while non‐normally distributed continuous variables were compared with the Mann‐Whitney U test. Proportions of binary data were compared using the Fisher's exact test. Z‐scores of plasma biomarker concentrations were also computed using the overall cohort mean and SD.

Differences in plasma biomarker concentrations between CAA and healthy controls, adjusting for age and sex were evaluated using linear regression. Assumptions of linear regression were evaluated and in instances where non‐normality of residuals or heteroscedasticity were encountered, log transformations were applied. Binary logistic regression models were constructed to evaluate biomarker predictivity, individually and in combination, of a diagnosis of CAA accounting for age and sex. Areas under the receiver operating characteristic (ROC) curve (AUC) were computed from logistic regression models to evaluate the diagnostic discriminability. AUCs of each model were compared using the test of equality of ROC areas to ascertain whether age and sex significantly improved discriminability. If an individual biomarker was significantly associated with CAA status, then it was considered for multivariable models. The Youden's Index was computed to ascertain optimal cut‐points, and we report their corresponding sensitivities and specificities.

## 3 RESULTS

### 3.1 Cohort characteristics

Overall, 92 of 120 participants had available plasma for analysis (45 CAA and 47 healthy controls). The reasons for missing samples were that samples were collected but exhausted as part of previous studies, or that there was human error in sample collection or processing. Table 1 summarizes differences in demographic, vascular risk factor and neuroimaging variables. The first clinical presentation of CAA included lobar ICH more than 90 days prior in 16 (35.6%), TFNEs in 17 (37.8%), cognitive impairment in 10 (22.2%) and CAA‐related inflammation in 2 (4.4%; these participants were studied while in remission and not taking immunosuppressants).

**TABLE 1:** Demographic, clinical, neuroimaging, and plasma biomarker characteristics of participants with CAA and healthy controls.

Variable	CAA (*n* = 45)	Healthy controls (*n* = 47)	*p*‐value
**Demographics**			
Age (years), mean (sd)	74.25 (7.18)	69.80 (6.43)	***p* = 0.002^a^**
Sex, female	18 (40.0%)	32 (68.1%)	***p* = 0.01^b^**
Years of education, median (IQR)	15 (12, 16)	16 (13.5,17)	*p* = 0.08^c^
**Vascular risk factors**			
Hypertension	27 (60.0%)	13 (27.7%)	***p* = 0.003 ^b^**
Dyslipidemia	20 (42.6%)	19 (42.2%)	*p* = 1.0 **^b^**
Diabetes	2 (4.4%)	4 (8.5%)	*p* = 0.36 **^b^**
Smoking history (any)	21/44 (47.7%)	17/44 (38.6%)	*p* = 0.52 **^b^**
**MRI markers of CAA**			
0 CMBs present	2/45 (4.44%)	41/45 (91.11%)	***p* < 0.0001 ^b^**
1 CMB present	4/45 (8.89%)	4/45 (8.89%)	
2 – 4 CMBs present	9/45 (20.0%)	0/45 (0.0%)	
≥ 5 CMBs Present	30/45 (66.7%)	0/45 (0.0%)	
Any cSS	28/45 (62.2%)	0/45 (0.0%)	***p* < 0.0001 ^b^**
Focal cSS	13/45 (28.9%)	0/45 (0.0%)	***p* < 0.0001 ^b^**
Disseminated cSS	15/45 (33.3%)	0/45 (0.0%)	***p* < 0.0001 ^b^**
High WMH	37/45 (82.2%)	12/45 (26.7%)	***p* < 0.0001 ^b^**
High CSO‐PVS count	37/45 (82.2%)	20/45 (44.4%)	***p* < 0.0001 ^b^**
Median CAA SVD score (IQR)	4 (4, 5)	1 (0, 1)	***p* < 0.0001 ^b^**
**Plasma biomarkers**			
Aβ_40_ (IPMS) pg/mL, median (IQR)	449.79 (411.44, 504.24) *n* = 41	444.63 (395.85, 472.38) *n* = 40	*p* = 0.27 ^c^
Aβ_42_ (IPMS) pg/mL, median (IQR)	40.84 (37.69, 44.60) *n* = 41	43.40 (38.45, 46.79) *n* = 40	*p* = 0.38 ^c^
Aβ_42/40_ (IPMS) ratio, mean (sd)	0.091 (0.008) *n* = 41	0.097 (0.009) *n* = 40	*p* = **0.002 ^a^**
Aβ_40_ (Simoa) pg/mL, median (IQR)	125.69 (105.03, 136.76) *n* = 36	119.53 (105.30, 134.06) *n* = 39	*p* = 0.67 ^c^
Aβ_42_ (Simoa) pg/mL, mean (sd)	6.44 (2.22) *n* = 36	7.76 (1.89) *n* = 39	*p* = **0.007 ^a^**
Aβ_42/40_ (Simoa) ratio, median (IQR)	0.056 (0.044, 0.060) *n* = 36	0.064 (0.060, 0.069) *n* = 39	***p* < 0.0001** ^c^
p‐tau‐181 pg/mL, median (IQR)	30.90 (22.44, 40.00) *n* = 36	20.03 (15.63, 24.87) *n* = 39	***p* < 0.0001** ^c^
GFAP pg/mL, median (IQR)	82.28 (43.72, 132.30) *n* = 36	79.17 (54.47, 108.45) *n* = 39	*p* = 0.92 ^c^
NfL pg/mL, median (IQR)	40.10 (23.83, 62.97) *n* = 36	17.86 (14.97, 23.19) *n* = 39	***p* < 0.0001** ^c^

*Note*: Superscripts indicate which statistical test was used: (a) Student's t‐test used when continuous variables were normally distributed (b) Fisher's exact chi‐squared test (c) Mann‐Whitney U Test when ordinal data compared or when distributions of continuous variables in either cohort were non‐normally distributed.

Abbreviations: Aβ, beta‐amyloid; CAA, cerebral amyloid angiopathy; CMBs, cortical microbleeds; cSS, cortical superficial siderosis; CSO‐PVS high centrum semiovale perivascular space count; GFAP, glial fibrillary acidic protein; ICH, Intracerebral Hemorrhage; IPMS, immunoprecipitation mass spectrometry; IQR, interquartile range; NfL, neurofilament light chain; p‐tau, phosphorylated tau; Simoa, single molecule array; TFNEs, transient focal neurological episodes; WMH, white matter hyperintensities.

Of the 92 samples sent to C_2_N, 10 had insufficient volume for analysis and 1 had interfering substances which prevented quantification. Specific characteristics of the participants with and without available plasma, those analyzed by IPMS (*n* = 81), Simoa (*n* = 75), and both (*n* = 66) are displayed in Tables S1‐S5 and patient flow diagrams are displayed in Figures S1 and S2.

### 3.2 Plasma biomarker differences

The mean (SD) or median (IQR) concentrations (pg/mL) of Aβ_40_, Aβ_42_, and the Aβ_42/40_ ratio, p‐tau‐181, GFAP, and NfL in CAA and healthy control groups are shown in Table 1.

### 3.3 Amyloid beta isoforms

**FIGURE 1:**
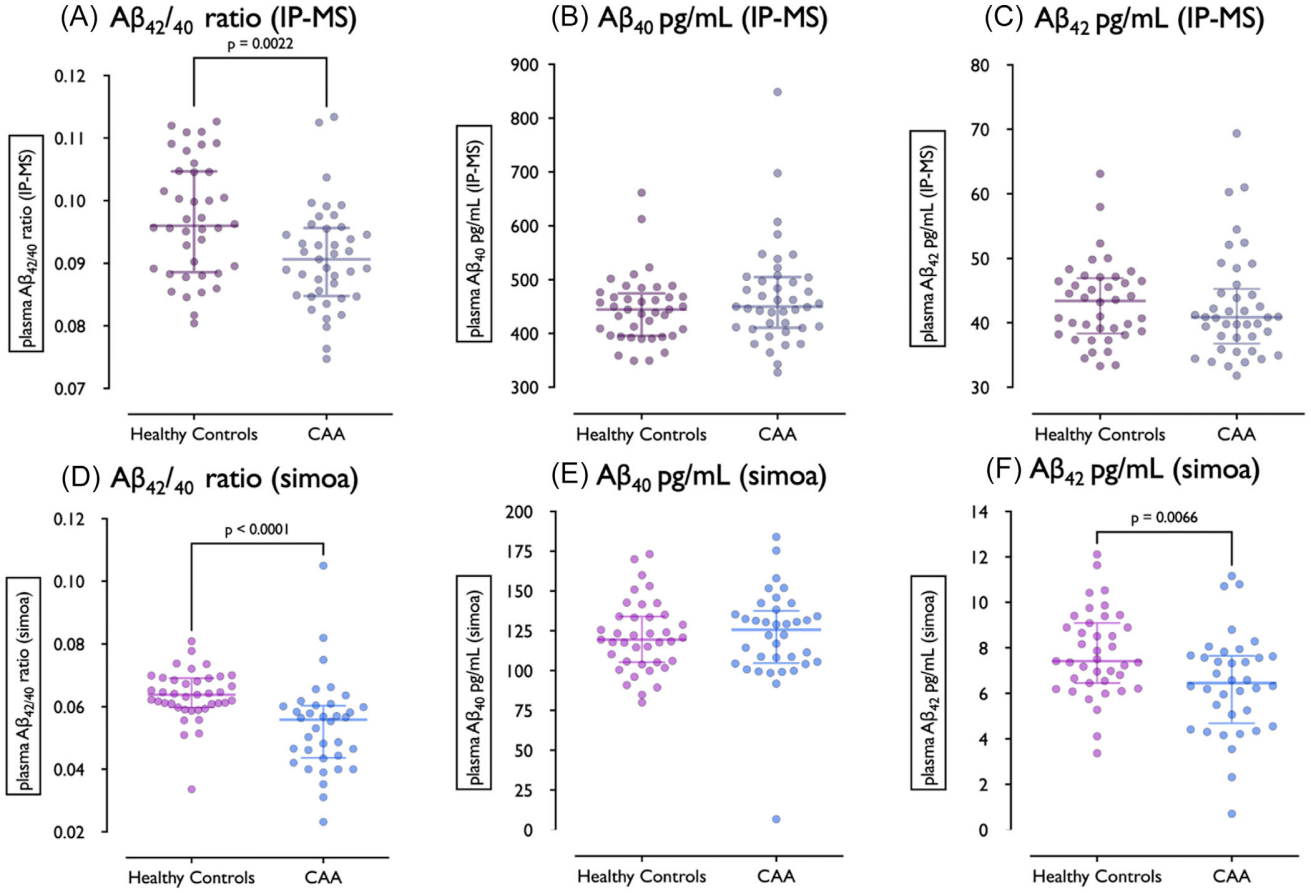
Dot plots of plasma concentrations of Aβ40, Aβ42, and the Aβ42/40 ratio quantified through IPMS (A‐C) and Simoa (D‐F) in healthy controls and CAA. Aβ, beta‐amyloid; CAA, cerebral amyloid angiopathy; IPMS, immunoprecipitation liquid chromatography mass‐spectrometry; Simoa, single molecule array.

The Aβ_42/40_ ratio was lower in CAA than healthy controls with both IPMS and Simoa. Simoa plasma Aβ_42_ was significantly lower in CAA than healthy controls. There were no differences in plasma Aβ_40_ using IPMS or Simoa. Concentrations of Aβ_40,_ Aβ_42,_ and the Aβ_42/40_ ratio are displayed in Table 1. Figure 1 depicts the dot plots of plasma Aβ concentrations. Overall, unadjusted Aβ_42/40_ (IPMS) and Aβ_42/40_ (Simoa) in CAA differed from healthy controls by ‐0.67 SD and ‐0.77 SD, respectively (Table 1 and Table S6).

### 3.4 p‐tau‐181, NfL, and GFAP

**FIGURE 2:**
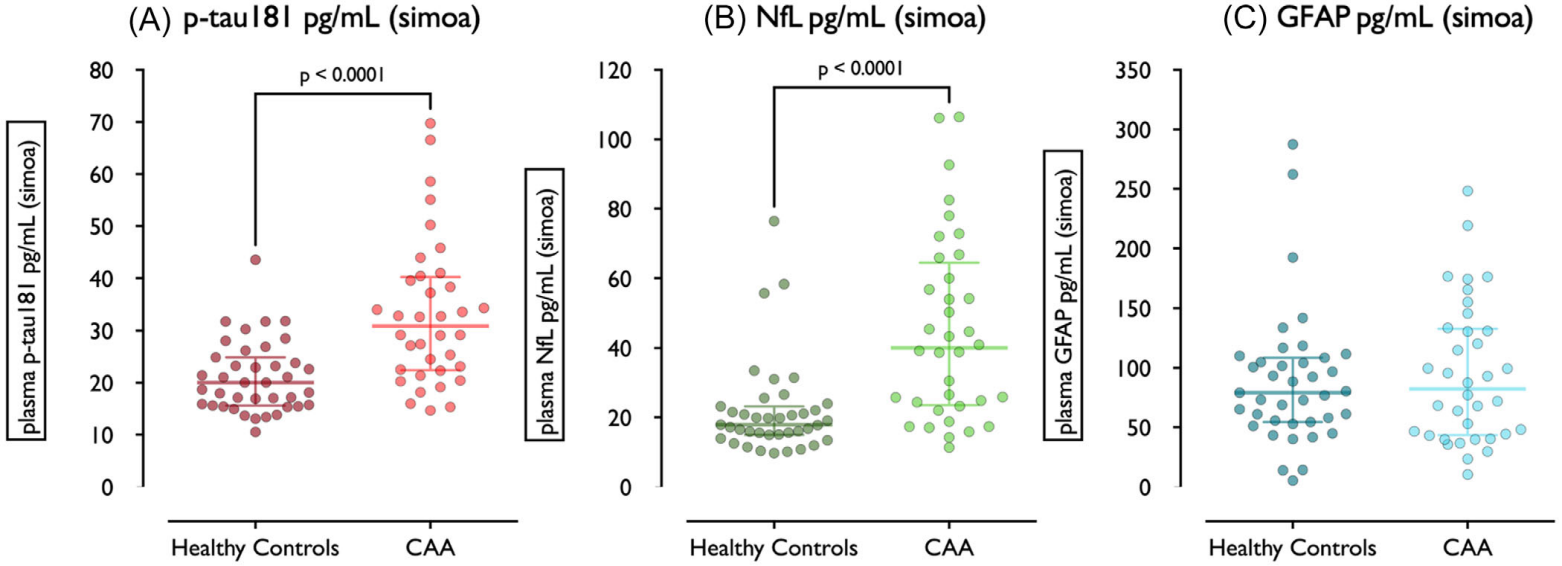
Dot plots of plasma concentrations of (A) p‐tau‐181, (B) NfL, and (C) GFAP quantified through Simoa in healthy controls and CAA. CAA, cerebral amyloid angiopathy; GFAP, glial fibrillary acidic protein; NfL, neurofilament light chain.

Participants with CAA had elevated median p‐tau‐181 and NfL compared to healthy controls. There was no statistically significant difference in plasma GFAP concentrations between CAA and healthy controls (Table 1, Figure 2). Overall, log(NfL) and log(p‐tau‐181) concentrations in CAA differed from healthy controls by +1.06 SD and +1.02 SD, respectively (Table S6).

### 3.5 Plasma biomarker differences adjusting for age and sex

Standardized beta‐coefficients of the z‐scores of Aβ_42/40_, log(GFAP), log(NfL), and log (p‐tau‐181), adjusted for age and sex, were generated in multilinear regression models. After adjustments for age and sex, significant differences in values of Aβ_42/40_ (Simoa) (β = ‐0.69, 95% CI: ‐1.15, ‐0.23, *p* = 0.004), NfL (β = 0.93, 95% CI: 0.54, 1.31, *p* < 0.0001), and p‐tau‐181 (β = 0.75, 95% CI: 0.35, 1.14, *p* < 0.0001) remained, but not for Aβ_42/40_ (IPMS) (β = ‐0.39, 95% CI: ‐0.83, 0.04, *p* = 0.08) (Table S6 **)**.

### 3.6 Validation against external normative reference intervals

Using age specific reference intervals from the Canada Health Measures Survey (*n* = 900),^33^ participants with CAA, compared to healthy controls, had Aβ_42/40_ (Simoa), p‐tau‐181 (trend), NfL, and GFAP levels that were more likely to fall outside of the population reference intervals (Table S7).^33^ Participants with CAA had a 9.17 (95% CI: 1.07, 78.77, *p* = 0.02) times greater odds than healthy controls of Aβ_42/40_ values occurring under the 5th percentile as well as 10.74 (95% CI: 2.79, 41.31, *p* = 0.0001) times greater odds of NfL values and 7.60 (95% CI: 0.87, 66.59, *p* = 0.036) times greater odds of p‐tau‐181 values falling above the 95th percentile compared to healthy controls (Table S8). There were no differences in the proportion of those with CAA versus healthy controls with GFAP values greater than the 95th percentile, however, those with CAA had a 3.84 (95% CI: 1.21, 12.25, *p* = 0.02) times greater odds of GFAP values occurring less than the 5th percentile compared to healthy controls (Table S9).

### 3.7 Predicting a diagnosis of cerebral amyloid angiopathy (Boston Criteria 2.0)

#### 3.7.1 Univariate logistic regression analyses

**FIGURE 3:**
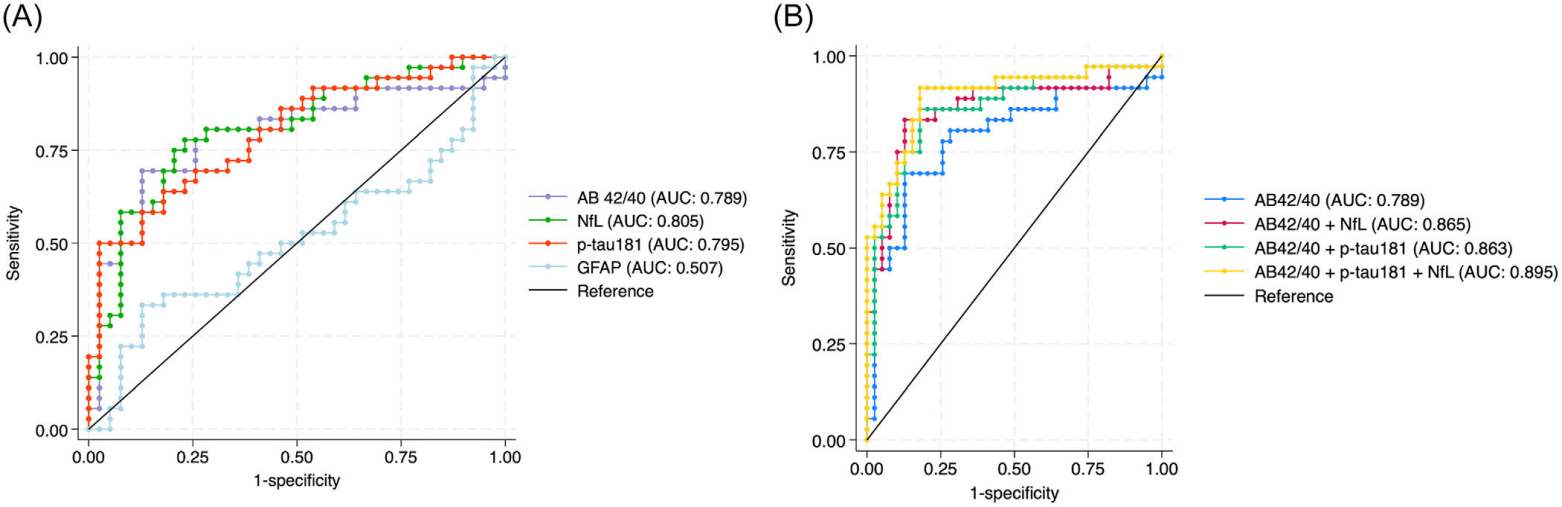
AUCs for Simoa plasma (A) Aβ_42/40_, p‐tau‐181, NfL, and GFAP individually and (B) in combination. Aβ, beta‐amyloid; AUC, total area under the receiver operating characteristic curve; GFAP, glial fibrillary acidic protein; NfL, neurofilament light chain; Simoa, single molecule array.

Table 2 displays the results of logistic regression models for individual biomarkers and Figure 3A depicts the individual ROCs for each Simoa biomarker. The Table 3 displays the sensitivities and specificities at the Youden's Index derived cut‐points for each plasma biomarker alone and in the models with subsequent age and sex adjustments.

**TABLE 2:** Summary of logistic regression models for individual biomarkers with standardized beta coefficients and AUC.

Biomarker	M	β log‐odds	β 95% CI	*p‐*Value	Sn	Sp	AUC (SE)	AUC 95% CI	ROC Comp
Aβ_42/40_ IPMS (Z‐score)	1^a^	−0.75	−1.26, ‐0.24	**0.004**	73.2%	62.5%	0.69 (0.06)	0.58, 0.79	*p* = 0.34
	1^b^	−0.67	−1.20, ‐0.14	**0.013**	78.0%	60.0%	0.74 (0.05)	0.63, 0.83	
	1^c^	−0.51	−1.08, 0.05	0.074	65.9%	75.0%	0.76 (0.05)	0.66, 0.85	
Aβ_42/40_ Simoa (Z‐score)	2^a^	−1.01	−1.65, ‐0.36	**0.002**	69.4%	87.2%	0.79 (0.06)	0.68, 0.87	*p* = 0.93
	2^b^	−0.90	−1.55, ‐0.26	**0.006**	75.0%	82.1%	0.79 (0.06)	0.68, 0.87	
	2^c^	−0.83	−1.47, ‐0.20	**0.01**	77.8%	76.9%	0.80 (0.05)	0.69, 0.88	
NfL (pg/mL) (Z‐score)	3^a^	1.49	0.67, 2.31	**<0.0001**	77.8%	76.9%	0.80 (0.05)	0.69, 0.88	*p* = 0.68
	3^b^	1.41	0.52, 2.31	**0.002**	75.0%	76.9%	0.80 (0.05)	0.69, 0.88	
	3^c^	1.50	0.57, 2.42	**0.002**	77.8%	79.5%	0.83 (0.05)	0.72, 0.90	
p‐tau‐181 (pg/mL) (Z‐score)	4^a^	1.59	0.74, 2.44	**<0.0001**	50.0%	97.4%	0.80 (0.05)	0.69, 0.88	*p* = 0.70
	4^b^	1.49	0.63, 2.36	**0.001**	75.0%	76.9%	0.80 (0.05)	0.69, 0.88	
	4^c^	1.40	0.50, 2.30	**0.002**	75.0%	79.5%	0.81 (0.05)	0.69, 0.88	
GFAP (pg/mL) (Z‐score)	5^a^	0.10	−0.36, 0.55	0.685	33.3%	87.2%	0.51 (0.07)	0.39, 0.62	*p* = **0.03**
	5^b^	−0.001	−0.48, 0.48	0.996	63.9%	71.8%	0.67 (0.07)	0.55, 0.77	
	5^c^	0.10	−0.41, 0.61	0.697	75.0%	64.1%	0.73 (0.06)	0.62, 0.83	

Abbreviations: Aβ, beta‐amyloid; AUC, total area under the receiver operating characteristic curve; CI, confidence interval; GFAP, glial fibrillary acidic protein; IPMS, immunoprecipitation liquid chromatography mass‐spectrometry; M, designates model number and superscripts indicated are as follows: a. unadjusted model, b. model adjusted for age, c. model adjusted for age and sex; NfL, neurofilament light chain; ROC Comp, Test of Equality of AUC areas; SE, standard error; Sn, sensitivity at the cut‐point, Sp, specificity at the cutpoint.

**TABLE 3:** Summary of logistic regression model receiver operating characteristic curves (AUC) for combinations of plasma biomarkers.

Parameter	Sn	Sp	AUC (SE)	AUC 95% CI	ROC Comp
**Aβ_42/40_ IPMS + Simoa biomarkers (*n* = 66)**					
Aβ_42/40_	73.2%	62.5%	0.66 (0.07)	0.52, 0.77	***p* = 0.01**
Aβ_42/40_ + NfL	75.8%	87.9%	0.86 (0.05)	0.76, 0.94	
Aβ_42/40_ + p‐tau‐181	48.5%	97.0%	0.77 (0.06)	0.65, 0.87	
Aβ_42/40_ + NfL + p‐tau‐181	78.8%	87.9%	0.87 (0.05)	0.76, 0.94	***p* =** 0.72
Aβ_42/40_ + NfL + p‐tau‐181 + age + sex	78.8%	87.9%	0.88 (0.04)	0.78, 0.95	
	75.8%	90.9%			
**Aβ_42/40_ Simoa + Simoa biomarkers (n=75)**					
Aβ_42/40_	69.4%	87.2%	0.79 (0.06)	0.68, 0.87	***p* =** 0.08
Aβ_42/40_ + NfL	83.3%	87.2%	0.87 (0.05)	0.77, 0.93	
Aβ_42/40_ + p‐tau‐181	86.1%	82.1%	0.86 (0.05)	0.77, 0.93	
Aβ_42/40_ + NfL + p‐tau‐181	91.7%	82.1%	0.90 (0.04)	0.80, 0.95	***p* =** 0.71
Aβ_42/40_ + NfL + p‐tau‐181 + age + sex	86.1%	79.5%	0.90 (0.04)	0.82, 0.96	
**Simoa biomarkers without Aβ_42/40_ (n=75)**					
NfL + p‐tau‐181	88.9%	66.7%	0.84 (0.04)	0.74, 0.91	***p* =** 0.12^*^

*Note*: Where ties occurred a single Youden's Index could not be computed and thus two probability cut‐points are reported with maximal correctly classified participants.

Abbreviations: Aβ, beta‐amyloid; AUC, total area under the receiver operating characteristic curve; NfL, neurofilament light chain; ROC Comp, Test of Equality of AUC areas; Simoa, single molecule array; Sn, sensitivity at the Youden's Index defined cut‐point; Sp, specificity at the Youden's Index defined cutpoint.

*This *p*‐value is for the test of equality of AUC areas comparing this model to the model Aβ_42/40_ + NfL + p‐tau‐181.

Individually, Aβ_42/40_ (IPMS), Aβ_42/40_ (Simoa), NfL, and p‐tau‐181 all predicted a diagnosis of CAA with AUCs of 0.69 for Aβ_42/40_ (IPMS) (95% CI: 0.58, 0.79, cut‐point: 0.095), 0.79 for Aβ_42/40_ (Simoa) (95% CI: 0.68, 0.87, cut‐point: 0.059); 0.81 for NfL (95% CI: 0.69, 0.88, cut‐point: 23.22pg/mL), and 0.80 for p‐tau‐181 (95% CI: 0.69, 0.88, cut‐point: 32.24 pg/mL). The addition of age and sex to these models did not increase their discriminative performance. The most specific single biomarker was p‐tau‐181 with a specificity of 97.4% and sensitivity of 50.0%, while the most sensitive single biomarker was NfL with a sensitivity of 77.8% and specificity of 76.9%. Aβ_42/40_ had intermediate sensitivities and specificities. GFAP alone did not discriminate a diagnosis of CAA from healthy controls.

### 3.8 Multivariable logistic regression analyses

Multiple biomarkers were evaluated in multivariable logistic regression models to compute AUCs, Youden's Index derived probability cut‐points, and corresponding sensitivities and specificities at these cut‐points (Table 3). The addition of NfL and p‐tau‐181 to a base model with Aβ_42/40_ (IPMS) improved the discriminative performance (*p* = 0.01), but the addition of NfL and p‐tau‐181 to a base model with Aβ_42/40_ (Simoa) did not (*p* = 0.08).

Overall convergent results were noted across the Aβ_42/40_ methods used. In the subset with both Aβ_42/40_ (IPMS) and Simoa based p‐tau‐181 and NfL, these plasma biomarkers predicted a diagnosis of CAA with an AUC of 0.87 (95% CI: 0.76, 0.94), with a sensitivity of 78.8% and specificity of 87.9% at the probability cut‐point.

In the subset with Simoa measured biomarkers, combining Aβ_42/40_, p‐tau‐181, and NfL yielded the most sensitive combination of biomarkers and predicted a diagnosis of CAA with an AUC 0.90 (95% CI: 0.80, 0.95), with a sensitivity of 91.7% and specificity of 82.1% at the probability cut‐point. The most specific combination of biomarkers, on the other hand, was observed with Aβ_42/40_ (IPMS) and p‐tau‐181 (sensitivity: 48.5%, specificity: 97.0%). The ROCs of the multivariable logistic regression models with Simoa based biomarkers are displayed in Figure 3B. The addition of age and sex to these models did not increase their discriminative performance (*p* ≥0.7). Table S10 depicts the logistic regression model log‐odds beta‐coefficients along with cut‐points derived from Youden's Index.

### 3.9 Subgroup analysis

We ran an additional subgroup analysis by excluding those probable CAA cases based on on an initial presentation with cognitive impairment (*n* = 10). As displayed in Table S11, we observed the same differences between those with CAA and healthy controls: reduced AB_42/40_ ratios with IPMS and Simoa as well as elevations in p‐tau‐181 and NfL, but not GFAP. Two multivariable logistic regression models were re‐fit, excluding those CAA participants diagnosed based on cognitive impairment. A model with AB_42/40_ (IPMS), NfL, and p‐tau‐181 discriminated a diagnosis of CAA from HC with an AUC = 0.86 (95% CI: 0.74, 0.93) (*n* = 61). A model with AB_42/40_ (Simoa), NfL, and p‐tau‐181 discriminated a diagnosis of CAA from HC with an AUC = 0.88 (95% CI: 0.78, 0.95) (*n* = 68).

## 4 DISCUSSION

Participants with Boston Criteria 2.0 defined probable CAA had reduced plasma Aβ_42/40_ ratios and elevated p‐tau‐181 and NfL concentrations compared to healthy controls. A combination of Aβ_42/40_ (Simoa), p‐tau‐181, and NfL resulted in the highest AUC of 0.90 (95% CI: 0.80, 0.95) and a corresponding sensitivity and specificity of 91.7% and 82.1%. The diagnostic test performance of these three plasma biomarkers is considered excellent.

These data raise some interesting pathophysiologic questions about CAA. The differences in plasma biomarkers we observed between CAA and healthy controls is a pattern of change that may also occur with AD, except that studies have reported elevations in GFAP in AD,^34^, ^35^ while we found that GFAP was not different in CAA. While our participants do have probable CAA, we cannot exclude the possibility of some degree of concomitant AD pathology. However, excluding ten CAA participants who presented with cognitive decline and therefore may be more likely to have concomitant AD did not change the study conclusions. Asymptomatic cerebral amyloidosis is common with increasing age,^15^ and since we did not screen our controls for amyloidosis before enrollment it is possible that intermediate sensitivities and specificities noted with Aβ_42/40_ could be related to asymptomatic amyloidosis among controls, which could bias our results towards null differences.

Elevations in p‐tau‐181 in our study may be because of concomitant AD with CAA, or may be due to direct effects of CAA on tau levels. Plasma p‐tau is also an indicator of cerebral amyloid deposition.^36^ Our plasma results are concordant with those from CSF, where patients with CAA have elevated CSF t‐tau and p‐tau compared to controls.^37^ Neuropathological evidence suggests that tau accumulation occurs in the neurites proximal to vessels affected by CAA.^38^, ^39^ One neuropathological study found that the correlation between tau‐tangles and amyloid was higher for amyloid plaque pathology (r = 0.77) than amyloid angiopathy (r = 0.32).^40^ Another study observed that greater severities of both CAA and plaque pathology correlated with higher tau pathology, suggesting that tau burden may jointly depend on both CAA and plaque pathology.^41^

Elevations in GFAP have been observed in AD and might be due to reactive astrocytosis to parenchymal amyloid plaque.^42^ In a neuropathological study, plasma GFAP was elevated in those with greater neuritic plaque pathology, but not with CAA.^43^ Comparable plasma concentrations of GFAP between CAA and healthy controls observed in our cohort might be related to a small sample size, but does generate several hypotheses: (1) there may be a lower burden of amyloid plaque in our CAA cohort (2) astrocytosis may not be as prominent feature of CAA; (3) there may be impaired clearance of GFAP into systemic circulation in CAA; or (4) there may be retreat of glial end feet from vessels afflicted with CAA. Larger studies are needed in the future to evaluate plasma GFAP in CAA compared to AD.

There are some important differences and similarities with respect to our findings in plasma and studies of CSF in CAA. A meta‐analysis of CSF biomarkers noted elevations in t‐tau, reductions in Aβ_42_ and Aβ_40_, and marginal differences with p‐tau in CAA compared to controls.^44^ Compared with AD, patients with CAA had lower Aβ_40_, similar Aβ_42_, lower p‐tau and lower t‐tau.^44^ One cohort study of CAA (*n* = 67), AD (*n* = 76), mild cognitive impairment (MCI) related to AD (*n* = 75), MCI unrelated to AD (*n* = 76), and controls (*n* = 78) noted the following patterns of CSF biomarker profiles: (1) reductions in CSF Aβ_40_ in CAA compared to controls, AD and MCI‐AD, and (2) elevations in t‐tau and p‐tau in CAA compared to MCI‐non‐AD and controls, but lower than AD and MCI‐AD.^45^ In plasma we did not observe differences between CAA and healthy controls in Aβ_40_ using either Simoa or IPMS methodology. Ultimately, CSF Aβ_40_ may be a more accurate reflection of central nervous system CAA than plasma Aβ_40_. However, we did find that adding Aβ_42/40_ to a model with p‐tau‐181 and NfL increased the specificity from 67% to 82%, suggesting that measuring plasma Aβ_42/40_ does have utility.

One review did not find differences in either plasma Aβ_40_ or Aβ_42_ in those with CAA compared to controls, but there was heterogeneity across plasma quantification methods and the Aβ_42/40_ ratio was not examined.^46^ In a large cohort study of participants with CAA related‐ICH, 95 healthy controls were compared to participants with probable CAA related acute ICH (n = 68) and, similar to our study, using Simoa methodology, elevations in plasma NfL and a reduction in the plasma Aβ_42/40_ ratio were noted.^47^ Overall, a small number of studies have evaluated plasma biomarkers in sporadic and autosomal dominant CAA with variable quantification methods and results.^48^, ^49^, ^50^, ^51^, ^52^

A strength of our study is that we corroborated the observations of the Aβ_42/40_ ratio using two validated modern methodologies: Simoa and IPMS. Furthermore, we leverage the existence of reference intervals for Simoa plasma biomarkers in the Canadian population to show that those with CAA, compared to healthy controls, had a 7.6 to 10.7 times greater odds of having plasma Aβ_42/40_, p‐tau‐181, and NfL values falling outside of reference intervals. This supports our study's external validity.

Limitations of this study include the small sample size, lack of external validation, and absence of an AD comparison group. Furthermore, including participants with a neuropathological diagnosis of CAA is needed to confirm the findings. For initial discovery, we used the Boston Criteria 2.0 as a reference standard, which has a sensitivity of 79.8% and specificity of 84.7% compared to neuropathologically confirmed CAA.^4^ Some of the apparent false positive and false negatives in this study may be due to the imperfect accuracy of the Boston Criteria 2.0, rather than limitations of the plasma assays, or possibly due to concomitant AD. Future analyses should also consider including apolipoprotein E (APOE) genotype in predicting CAA.^53^ Finally, the previously published external normative reference intervals were run on a different lot and, thus, lot variability may be a source of bias.^33^ Future studies should compare plasma biomarker profiles between those meeting diagnostic criteria for probable CAA by Boston Criteria 2.0 but not version 1.5, as these individuals would meet probable CAA on the basis of a single lobar hemorrhagic lesion only plus the added supportive white matter features, and may be at an earlier stage of CAA.^15^ Future studies should also directly compare plasma biomarkers between CAA, AD, and non‐CAA ICH cohorts and determine whether plasma markers can predict recurrent ICH. Additionally, studies are needed to compare plasma biomarkers in the different CAA clinical syndromes, including in patients that present with cognitive decline only, without history of hemorrhagic stroke.

Overall, this study suggests that plasma Aβ_42/40_, p‐tau‐181, and NfL together can discern Boston Criteria 2.0 probable CAA from healthy controls. Compared to PET and CSF studies, plasma is more accessible and scalable. Identifying a plasma biomarker signature of CAA may advance the diagnostic discriminability beyond clinical and imaging criteria and may also facilitate the diagnostic workup for those with ICH and those who cannot undergo MRI.

Our study also highlights the need for investigations of biomarkers in larger cohorts of CAA, which may be expeditiously leveraged by international collaborative working groups with pooled test and validation cohorts. Considering the recent advances in AD beta‐amyloid targeting immunotherapies and the concern raised for a higher risk of ARIA among those with concomitant CAA, identifying a distinguishing plasma biomarker panel of CAA from AD will be of paramount importance in future.^12^, ^13^, ^19^

## CONFLICTS OF INTEREST STATEMENT

R.T.M.: does not have any perceived or actual conflicts of interest relevant to this work. S.S.: does not have any perceived or actual conflicts of interest relevant to this work. J.G.C.: does not have any perceived or actual conflicts of interest relevant to this work. A.E.B.: does not have any perceived or actual conflicts of interest relevant to this work. C.R.M.: does not have any perceived or actual conflicts of interest relevant to this work. M.G.: does not have any perceived or actual conflicts of interest relevant to this work. K.N.: does not have any perceived or actual conflicts of interest relevant to this work. N.N.: does not have any perceived or actual conflicts of interest relevant to this work. J.V.: does not have any perceived or actual conflicts of interest relevant to this work. K.M.K.: is employed by C2N Diagnostics and owns equity shares in C2N Diagnostics. S.E.B.: Contract Research: GE Healthcare, Genentech, Optina, Roche, Eli Lilly, Eisa/Biogen Idec, NovoNordisk, Lilly Avid. Payments made to Institution. No personal investigator fees taken including Eli Lilly. Peer Reviewed: Ontario Brain Institute, CIHR, Leducq Foundation, Heart and Stroke Foundation of Canada, NIH, Alzheimer's Drug Discovery Foundation, Brain Canada, Weston Brain Institute, Canadian Partnership for Stroke Recovery, Canadian Foundation for Innovation, Focused Ultrasound Foundation, Alzheimer's Association US, Department of National Defence, Montreal Medical International Kuwait, Queen's University, Compute Canada Resources for Research Groups, CANARIE, Networks of Centres of Excellence of Canada. Payments made to Institution. No personal investigator fees taken. Consulting Fees: Roche, Biogen, Eli Lilly, NovoNordisk, Eisai. Payments made to me. Honoraria: Biogen, Roche Models of Care Analysis in Canada, and Eisai MRI Workshop. Payments made to me. M.D.H.: Consulting fees: Sun Pharma, Brainsgate Inc (paid work for adjudication of clinical trial outcomes); Grants/Contracts: ESCAPE‐NA1 and ESCAPE NEXT trials (NoNO Inc. CIHR), ESCAPE MeVO study (Medtronic); TEMPO‐2 trial (CIHR, Heart & Stroke Foundation of Canada, Boehringer‐Ingelheim). Consulting Fees: Sun Pharmam Brainsgate Inc (paid work for adjudication of clinical trial outcomes). Stock or stock options: Circle Inc. Leadership or fiduciary role: President (Canadian Neurological Sciences Federation (not for profit), Canadian Stroke Consortium (not for profit). Data Safety Monitoring Board activity: DSMC Chair (DUMAS trial, Oncovir Hiltonel trial) and DSMB member (ARTESIA trial, BRAIN‐AF trial, LAAOS‐4 trial). R.C.: Grants: CIHR, Canadian Consortium on Neurodegeneration of Aging and Compass ND/CIHR; Sparx3/NIH, Synergic‐2 Clinical Trial (Weston Foundation to University of Alberta via Western University). Monitoring Board activity: Amborxal Trial (unpaid scientific advisor) and Parkinson Canada (unpaid research and clinical advisory board). C.L.W.: does not have any perceived or actual conflicts of interest relevant to this work. E.E.S.: has previously served on a steering committee (unpaid) for Alnylam Pharmaceuticals and on advisory boards (unpaid) for Eisai and Eli Lilly. Author disclosures are available in the Supporting Information.

## CONSENT STATEMENT

All human participants provided informed written consent to participate in this research study.

## Supporting information



Supporting Information

Supporting Information

Supporting Information

Supporting Information

## Data Availability

Anonymized data will be made available to other qualified researchers on reasonable request to the corresponding author for the purposes of replicating analyses.
