# Supplementary figures and images for: Plasma biomarkers distinguish Boston Criteria 2.0 cerebral amyloid angiopathy from healthy controls
Source: Alzheimers Dement. 2025 Mar 29;21(3):e70010. doi: 10.1002/alz.70010 (PMC11953569; doi:10.1002/alz.70010)

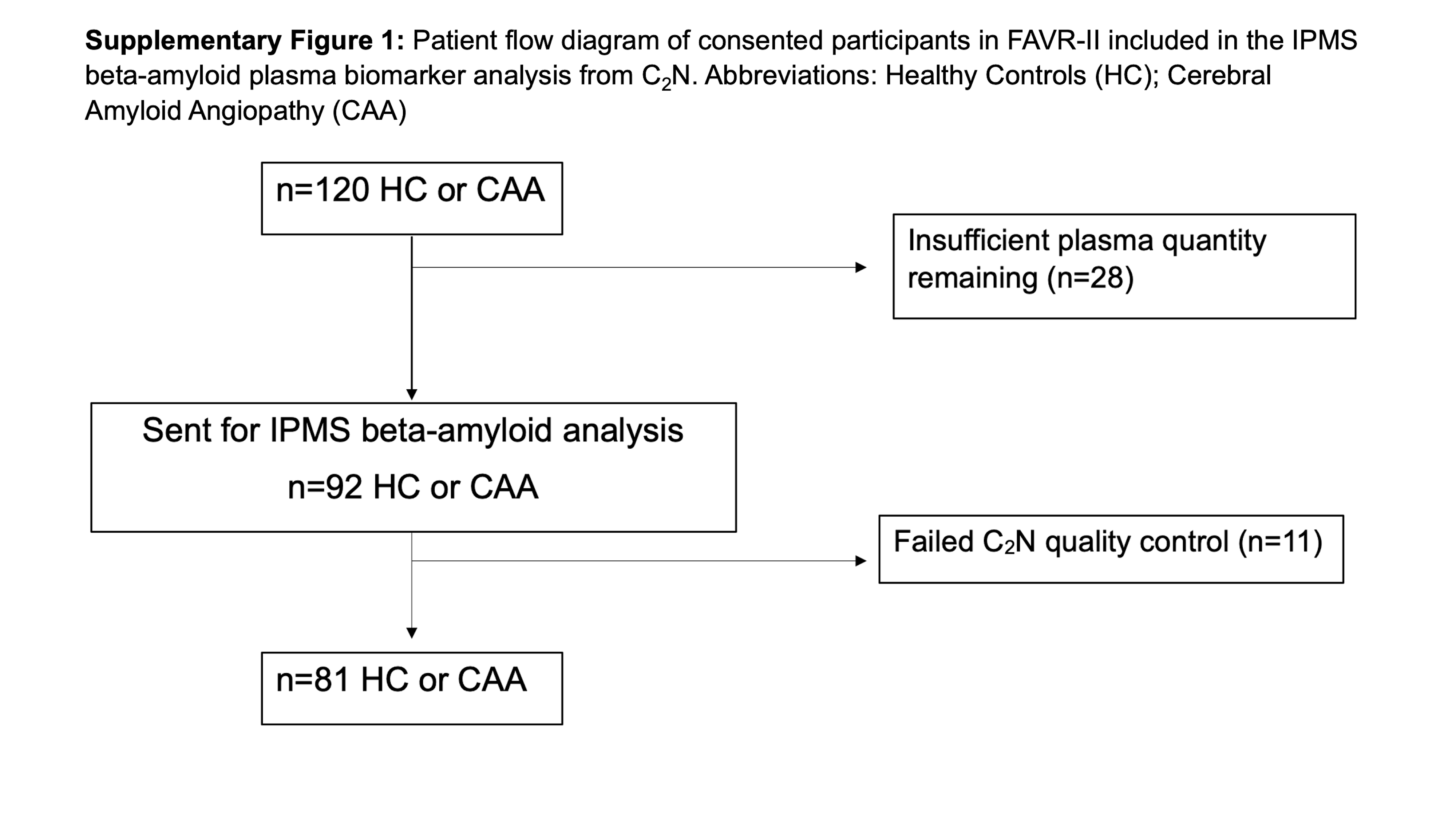

Supplement: Supplementary file 1 — Supporting Information [file ALZ-21-e70010-s003.tiff]

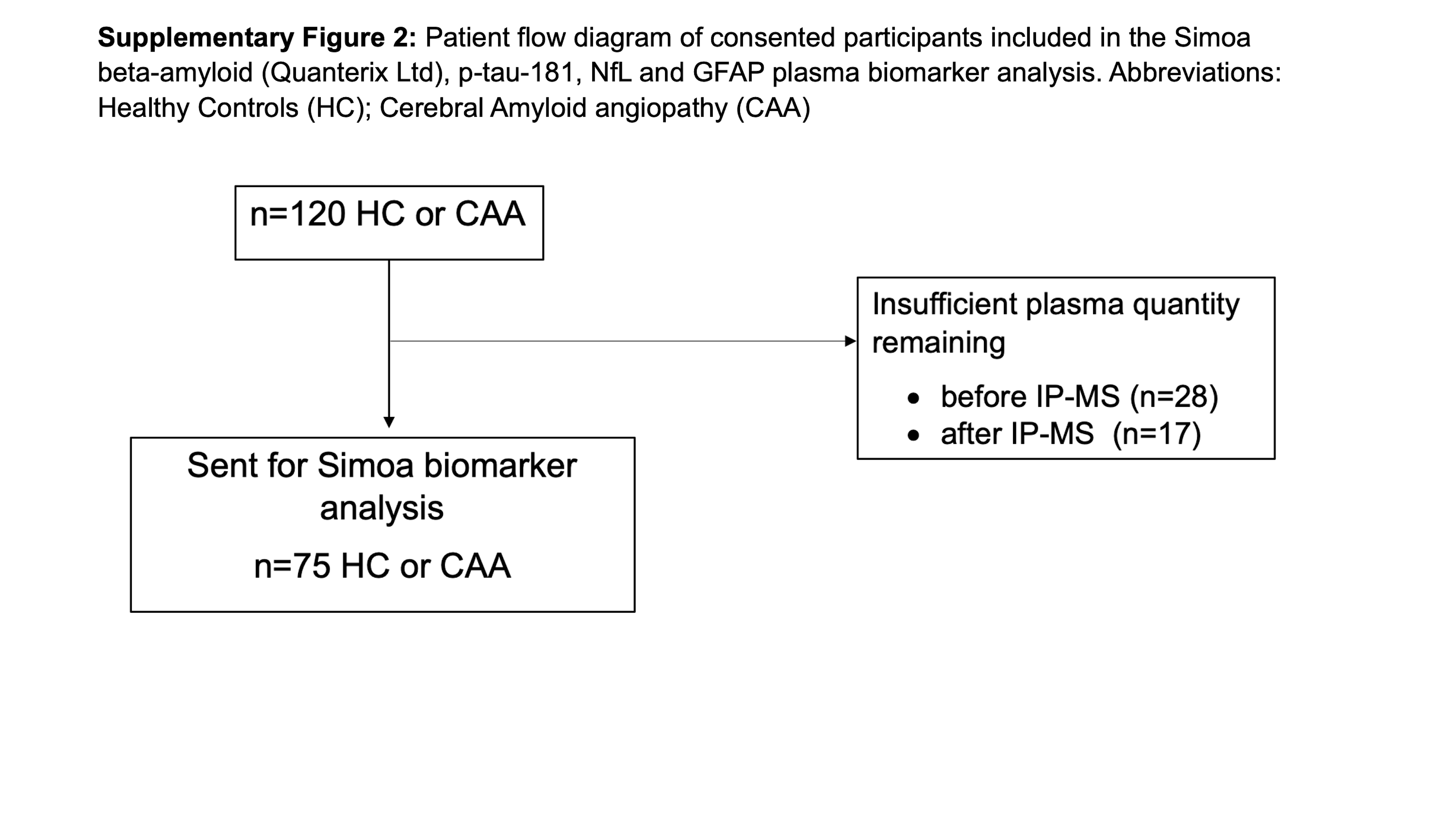

Supplement: Supplementary file 2 — Supporting Information [file ALZ-21-e70010-s002.tiff]
