# Supplementary material for: Plasma biomarkers distinguish Boston Criteria 2.0 cerebral amyloid angiopathy from healthy controls
Source: Alzheimers Dement. 2025 Mar 29;21(3):e70010. doi: 10.1002/alz.70010 (PMC11953569; doi:10.1002/alz.70010)
Supplement: Supplementary file 3 — Supporting Information [file ALZ-21-e70010-s001.pdf]

ICMJE DISCLOSURE FORM

**Date:**	11/17/2024
**Your Name:**	Sandra E. Black
**Manuscript Title:**	Plasma biomarkers distinguish Boston Criteria 2.0 cerebral amyloid angiopathy from healthy controls
**Manuscript Number (if known):**	ADJ-D-24-01749

In the interest of transparency, we ask you to disclose all relationships/activities/interests listed below that are related to the content of your manuscript. "Related" means any relation with for-profit or not-for-profit third parties whose interests may be affected by the content of the manuscript. Disclosure represents a commitment to transparency and does not necessarily indicate a bias. If you are in doubt about whether to list a relationship/activity/interest, it is preferable that you do so.

The author's relationships/activities/interests should be defined broadly. For example, if your manuscript pertains to the epidemiology of hypertension, you should declare all relationships with manufacturers of antihypertensive medication, even if that medication is not mentioned in the manuscript.

In item #1 below, report all support for the work reported in this manuscript without time limit. For all other items, the time frame for disclosure is the past 36 months.

		**Name all entities with whom you have this relationship or indicate none (add rows as needed)**	**Specifications/Comments (e.g., if payments were made to you or to your institution)**
		Time frame: Since the initial planning of the work	
1	All support for the present manuscript (e.g., funding, provision of study materials, medical writing, article processing charges, etc.)<br>No time limit for this item.	☒ None	
			Click the tab key to add additional rows.
		Time frame: past 36 months	
**2**	Grants or contracts from any entity (if not indicated in item #1 above).	☐ **None**	
		Contract Research: Genentech, Optina, Roche, Eli Lilly, Eisa/Biogen Idec, NovoNordisk, Lilly Avid, ICON, Aribio Co.	Payments made to Institution.<br>No personal investigator fees taken including Eli Lilly.
		Peer Reviewed: Ontario Brain Institute, CIHR, Leducq Foundation, Heart and Stroke Foundation of Canada, NIH, Alzheimer's Drug Discovery Foundation, Brain Canada, Weston Brain Institute, Canadian Partnership for Stroke Recovery, Canadian Foundation for Innovation, Focused Ultrasound Foundation, Alzheimer's Association US, Queen's University, Compute Canada Resources for Research Groups,	Payments made to Institution.<br>No personal investigator fees taken.

		Name all entities with whom you have this relationship or indicate none (add rows as needed)	Specifications/Comments (e.g., if payments were made to you or to your institution)
		CANARIE, Networks of Centres of Excellence of Canada	
3	Royalties or licenses	☒ **None**	
4	Consulting fees	☐ **None**	
		Roche	Payments made to me
		Biogen	Payments made to me
		NovoNordisk	Payments made to me
		Eisai	Payments made to me
		Eli Lilly	Payments made to me
		DSR: Diagnosis, Solutions & Results Inc.	Payment made to institution (CBRR)
5	Payment or honoraria for lectures, presentations, speakers bureaus, manuscript writing or educational events	☐ **None**	
		Biogen	Payments made to me
		Roche New England Journal Manuscript	No Payments to me
		Roche Models of Care Analysis in Canada in Submission	Payments made to me for advising and manuscript review
		Eisai MRI Workshop	Payments made to me
		Cpdnetwork planning committee for AD educational program	Payments made to me
6	Payment for expert testimony	☒ **None**	
7	Support for attending meetings and/or travel	☒ **None**	
8	Patents planned, issued or pending	☒ **None**	

		Name all entities with whom you have this relationship or indicate none (add rows as needed)	Specifications/Comments (e.g., if payments were made to you or to your institution)
**9**	Participation on a Data Safety Monitoring Board or Advisory Board	☐ **None**	
		Conference Board of Canada<br>World Dementia Council<br>University of Rochester Contribution to the Mission and Scientific Leadership of the Small Vessel VCID Biomarker Validation Consortium<br>National Institute of Neurological Disorders and Stroke, Ontario Dementia Care Alliance (ODCA)	Advisory Boards only.<br>No personal fees taken.
**10**	Leadership or fiduciary role in other board, society, committee or advocacy group, paid or unpaid	☒ **None**	
**11**	Stock or stock options	☒ **None**	
**12**	Receipt of equipment, materials, drugs, medical writing, gifts or other services	☒ **None**	
**13**	Other financial or non-financial interests	☒ **None**	

Please place an "X" next to the following statement to indicate your agreement:

☒ I certify that I have answered every question and have not altered the wording of any of the questions on this form.
