# Supplementary material for: Plasma biomarkers distinguish Boston Criteria 2.0 cerebral amyloid angiopathy from healthy controls
Source: Alzheimers Dement. 2025 Mar 29;21(3):e70010. doi: 10.1002/alz.70010 (PMC11953569; doi:10.1002/alz.70010)
Supplement: Supplementary file 4 — Supporting Information [file ALZ-21-e70010-s004.docx]

**DATA SUPPLEMENT**

**Plasma biomarkers distinguish Boston Criteria 2.0 cerebral amyloid angiopathy from healthy controls**

Ryan T. Muir^1,2,3,5^, Sophie Stukas,^6^ Jennifer G. Cooper,^6^ Andrew E. Beaudin^2,5^, Cheryl R. McCreary^2,4,5^, Myrlene Gee,^7^ Krista Nelles^7^, Nikita Nukala^5^, Janina Valencia^5^, Kristopher M Kirmess^9^, Sandra E. Black^10^, Michael D. Hill,^1,2,3^ Richard Camicioli,^7,8^ Cheryl L. Wellington^6^, Eric. E Smith^1,2,3,5^

Calgary Stroke Program; 2. Hotchkiss Brain Institute; Departments of 3. Community Health Sciences 4. Radiology and 5. Clinical Neurosciences, University of Calgary, Calgary, 2500 University Dr NW, Calgary, Alberta, Canada T2N 1N4, Canada. 6. Department of Pathology and Laboratory Medicine, Djavad Mowafaghian Centre for Brain Health, University of British Columbia, 2215 Wesbrook Mall, Vancouver, British Columbia, V6T 1Z3, Canada. 7. Division of Neurology, Department of Medicine 8. Neuroscience and Mental Health Institute, University of Alberta, 16 St & 85 Ave, Edmonton, Alberta, T6G 2R3, Canada. 9. C2N Diagnostics, 4340 Duncan Ave, St. Louis, MO, 63110, United States 10. L.C Campbell Cognitive Neurology Research Unit, Dr Sandra Black Centre for Brain Resilience and Recovery, and Hurvitz Brain Sciences Program, Sunnybrook Research Institute, University of Toronto, 2075 Bayview Ave, North York, Ontario M4N 3M5, Canada.

Table of Contents

[Supplementary Methodology 3](#_Toc176360854)

[Supplementary Tables 5](#_Toc176360855)

[Supplementary Table 1: 5](#_Toc176360856)

[Supplementary Table 2: 7](#_Toc176360857)

[Supplementary Table 3: 9](#_Toc176360858)

[Supplemental Table 4: 11](#_Toc176360859)

[Supplemental Table 5: 13](#_Toc176360860)

[Supplemental Table 7: 15](#_Toc176360861)

[Supplemental Table 8: 17](#_Toc176360862)

[Supplemental Table 9: 18](#_Toc176360863)

[Supplemental Table 10: 19](#_Toc176360864)

[SUPPLEMENTARY FIGURES: 20](#_Toc176360865)

[Supplementary Figure 1: 20](#_Toc176360866)

[Supplementary Figure 2: 20](#_Toc176360867)

# **Supplementary Methodology**

**Plasma Biomarker Quantification**

***Simoa***

Quality control measures were recorded, and all samples fell within the assay limits of quantification. There were 4 samples with high intra-sample coefficients of variability (CV) for p-tau-181 (CVs>50%) unrelated to machine or sample quality. These four samples did not pose a problem for the N4PE assay, which implied that there was not an overall problem with the sample. These were re-run and CVs<10.18% were achieved upon re-analysis. The values from the repeated analyses were valid and subsequently used.

While normative data from the ageing Canadian population as reported in Cooper et al.^1^ for plasma Aβ_42/40_ , GFAP, and NfL were based off the Neurology-4-plex E advantage assay (catalogue 103670, lot 503105) version 2.1 Advantage Kit (cat ID 104111, lot 503843) was used, the plasma p-tau-181 was measured using the p-tau-181 version 2.0 advantage assay (catalogue 103714, lot 502923). In the current analyses the version 2.1 Advantage Kit (catalogue #104111, lot 503843) was used. Therefore, to compare v2.1 p-tau-181 concentrations in the current analyses to the normative data from Cooper et al.^1^ a conversion factor published by Quanterix was applied. This conversion factor is published in a technical note by Quanterix entitled: Simoa p-tau 181 Advantage V2.1 Assay (reference TECH-0153), released specifically to describe the differences between the V2.0 and V2.1 assay. ^2^

**Statistical Analyses**

**Linear Regression:**

Multivariable linear regression models were constructed to evaluate for differences in plasma biomarkers between groups while adjusting for age and sex. The plasma biomarker values were transformed to Z scores by subtracting the group mean from each participant’s value and dividing this by the group standard deviation. In all models the assumptions of no multicollinearity, normality of residuals, and homoscedasticity were evaluated. Normality of residuals were evaluated with kernel density curves and q-q plots. Homoscedasticity was evaluated using Cameron & Trivedi's test for heteroskedasticity. Multi-collinearity was evaluated using Variance Inflation Factor. In instances where the assumptions of normality of residuals and/or homoscedasticity were violated, the plasma biomarker values were log transformed first then transformed to Z scores for modelling. The results of these models are reported below in **Supplementary Table 7.**

**Comparison to normative population data**

As the data provided in Cooper et al.^1^ apply to an age range of 3 to 79 years and our dataset had participants 80 and over, we used the 79 year-old reference values for the 9 participants age 80 and over. To define those <5^th^ percentile the lower bound 95% confidence interval value of the predicted age specific 5^th^ percentile value was used. To define those >95^th^ percentile the upper bound of the 95% confidence interval value of the predicted age specific 95^th^ percentile value was used. These data are displayed below in **Supplementary Table 7**.

The number of participants in the Normal Control and CAA cohorts with (A) within the range of normative values produced from Cooper et al were “Test Negative” and (B) out of range, “Test Positive,” abnormal was defined as (1) <5%tile for Aβ_42/40_ (2) >95%tile for p-tau-181, GFAP, and NfL. An odds ratio was then constructed using the Woolf method for estimating confidence intervals around the point estimate (as displayed in **Supplementary Table 7**).

One of the important limitations for using the population normative data from Cooper et al. ^1^, is that the the recruited n=900 participants were consenting Canadians between the ages of 3 – 79 years without any inclusion or exclusion criteria on the basis of cognitive impairment or the presence of neurologic disorders which can lead to cognitive impairment and/or dementia. It is likely that the normative population data from Cooper et al. contains persons with cognitive disorders and concomitant neurologic disorders (such as neurodegenerative diseases). Thus, the normative population data are not equivalent to our healthy control cohort where exclusion criteria were in place for cognitive impairment and concomitant neurological disorders.

# **Supplementary Tables**

**Supplementary Table 1:** Demographic, Vascular Risk Factor, Cognitive and Neuroimaging Data for participants with sufficient samples sent to C2N for IPMS quantification of plasma amyloid-beta.

| **Variable** | **CAA** (n=41) | **Healthy controls** (n=40) | **p-value** |
| --- | --- | --- | --- |
| **Demographics** | | | |
| Age (years), mean (sd) | 74.39 (7.46) | 69.58 (6.20) | ***p*=0.002^a^** |
| Sex, female | 18 (43.9%) | 27 (67.5%) | ***p*=0.045^b^** |
| Years of Education, mean (sd) | 14.37 (3.24) | 15.79 (3.28) | *p*=0.05^a^ |
| **Vascular Risk Factors** | | | |
| Hypertension | 23 (56.1%) | 10 (25.0%) | ***p*=0.006 ^b^** |
| Dyslipidemia | 15 (36.6%) | 16 (40.0%) | *p*=0.82 **^b^** |
| Diabetes | 1 (2.4%) | 3 (7.5%) | *p*=0.36 **^b^** |
| Smoking history (any) | 19/40 (47.5%) | 15/37 (40.5%) | *p*=0.65 **^b^** |
| **Initial CAA Presentation** | | | |
| Lobar ICH | 15 (36.6%) | - |  |
| TFNEs | 16 (39.0%) | - |  |
| Cognitive impairment | 8 (19.5%) | - |  |
| CAA-related inflammation | 2 (4.9%) | - |  |
| **MRI Markers of CAA** | | | |
| 0 – 1 CMBs Present | 6/41 (14.6%) | 38/38 (100%) | *p*<0.0001 ^b^ |
| 2 – 4 CMBs Present | 8/41 (19.5%) | 0/38 (0.0%) |  |
| ≥ 5 CMBs Present | 27/41 (65.9%) | 0/38 (0.0%) |  |
| Any cSS | 27/41 (65.9%) | 0/38 (0.0%) | *p*<0.0001 ^b^ |
| Focal cSS | 12/41 (29.3%) | 0/38 (0.0%) | *p*<0.0001 ^b^ |
| Disseminated cSS | 15/41 (36.6%) | 0/38 (0.0%) | *p*<0.0001 ^b^ |
| High WMH | 34/41 (82.9%) | 10/38 (26.3%) | *p*<0.0001 ^b^ |
| High CSO-PVS count | 33/41 (80.5%) | 17/38 (44.7%) | ***p*<0.0001 ^b^** |
| CAA SVD Score (median, IQR) | 4 (4, 5) | 1 (0, 1) | ***p*<0.0001 ^b^** |

**Abbreviations:** CAA, cerebral amyloid angiopathy; ICH, Intracerebral Hemorrhage; TFNEs, transient focal neurological episodes; IPMS, immunoprecipitation mass spectrometry; CMBs, cortical microbleeds; cSS, cortical superficial siderosis; WMH, white matter hyperintensities; CSO-PVS high centrum semiovale perivascular space count.. Superscripts indicate which statistical test was used: (a) Student’s t-test used when continuous variables were normally distributed (b) Fisher’s Exact Chi-Squared test (c) Mann-Whitney U Test when ordinal data compared or when distributions of continuous variables in either cohort were non-normally distributed.

**Supplementary Table 2:** Demographic, Vascular Risk Factor, Cognitive and Neuroimaging Data for participants with sufficient samples sent for Simoa quantification plasma amyloid-beta, p-tau-181, NfL, and GFAP.

| **Variable** | **CAA** (n=36) | **Healthy controls** (n=39) | **p-value** |
| --- | --- | --- | --- |
| **Demographics** | | | |
| Age (years), mean(sd) | 74.11 (7.29) | 70.39 (6.10) | ***p*=0.02** ^a^ |
| Sex, female | 16 (44.4%) | 27 (69.2%) | ***p*=0.04** ^b^ |
| Years of Education, median (IQR) | 15 (12.5, 17) | 15 (13.5, 17) | *p*=0.50 ^c^ |
| **Vascular Risk Factors** | | | |
| Hypertension | 21 (58.3%) | 10 (25.6%) | ***p*=0.005** ^b^ |
| Dyslipidemia | 14 (38.89%) | 16 (41.0%) | *p*=1.00 **^b^** |
| Diabetes | 1 (2.8%) | 3 (7.7%) | *p*=0.62 **^b^** |
| Smoking history (any) | 13/35 (37.1%) | 14/37 (37.8%) | *p*=1.00 **^b^** |
| **Initial CAA Presentation** | | | |
| Lobar ICH | 13 (36.1%) | - |  |
| TFNEs | 14 (38.9%) | - |  |
| Cognitive impairment | 7 (19.4%) | - |  |
| CAA-related inflammation | 2 (5.6%) | - |  |
| **MRI Markers of CAA** | | | |
| 0 – 1 CMBs Present | 5 (13.9%) | 38/38 (100.0%) | ***p*<0.0001**^b^ |
| 2 – 4 CMBs Present | 7 (19.4%) | 0/38 (0.0%) |  |
| ≥ 5 CMBs Present | 24 (66.7%) | 0/38 (2.6%) |  |
| Any cSS | 23 (63.9%) | 0/38 (0.0%) | ***p*<0.0001**^b^ |
| Focal cSS | 11 (30.6%) | 0/38 (0.0%) | ***p*<0.0001**^b^ |
| Disseminated cSS | 12 (33.3%) | 0/38 (0.0%) | ***p*<0.0001**^b^ |
| High WMH | 29 (80.6%) | 11/38 (29.0%) | ***p*<0.0001**^b^ |
| High CSO-PVS count | 30 (83.3%) | 19/38 (50.0%) | ***p*=0.003** ^b^ |
| CAA SVD Score (median, IQR) | 4 (4, 5) | 1 (0, 1) | ***p*<0.0001**^b^ |

**Abbreviations:** CAA, cerebral amyloid angiopathy; ICH, Intracerebral Hemorrhage; TFNEs, transient focal neurological episodes; Simoa, single molecule array; Aβ, amyloid beta; p-tau, phosphorylated tau; GFAP, glial fibrillary acidic protein; NfL, neurofilament light; CMBs, cortical microbleeds; cSS, cortical superficial siderosis, WMH, white matter hyperintensities; CSO-PVS high centrum semiovale perivascular space count. Superscripts indicate which statistical test was used: (a) Student’s t-test used when continuous variables were normally distributed (b) Fisher’s Exact Chi-Squared test (c) Mann-Whitney U Test when ordinal data compared or when distributions of continuous variables in either cohort were non-normally distributed.

**Supplementary Table 3:** Demographic, Vascular Risk Factor, Plasma Biomarker, Cognitive and Neuroimaging Data for participants who had both IPMS and Simoa plasma biomarker quantification.

| **Variable** | **CAA** (n=33) | **Healthy controls** (n=33) | **p-value** |
| --- | --- | --- | --- |
| **Demographics** | | | |
| Age (years), mean (sd) | 73.95 (7.59) | 70.09 (5.78) | ***p*=0.02^a^** |
| Sex, female | 16 (48.5%) | 22 (66.7%) | *p*=0.21^b^ |
| Years of Education, mean (sd) | 14.91 (3.15) | 15.80 (3.52) | *p*=0.28^a^ |
| **Vascular Risk Factors** | | | |
| Hypertension | 18 (54.6%) | 8 (24.2%) | ***p*=0.02^b^** |
| Dyslipidemia | 11 (33.3%) | 13 (39.4%) | *p*=0.80^b^ |
| Diabetes | 0 (0.0%) | 3 (9.1%) | *p*=0.24^b^ |
| Smoking history (any) | 12/32 (37.5%) | 12/31 (38.7%) | *p*=1.00^b^ |
| **Initial CAA Presentation** | | | |
| Lobar ICH | 12 (36.4%) | - |  |
| TFNEs | 14 (42.4%) | - |  |
| Cognitive impairment | 5 (15.2%) | - |  |
| CAA-related inflammation | 2 (6.1%) | - |  |
| **Plasma Biomarkers** | | | |
| Aβ_40_ pg/mL IPMS, median (IQR) | 446.31 (409.85, 495.35) | 436.33 (395.52, 466.92) | *p*=0.34 ^c^ |
| Aβ_42_ pg/mL IPMS, median (IQR) | 40.75 (35.87, 42.54) | 40.99 (38.11, 46.18) | *p*=0.42 ^c^ |
| Aβ_42/40_ ratio IPMS, mean (sd) | 0.092 (0.009) | 0.096 (0.009) | ***p*=0.035^a^** |
| Aβ_40_ pg/mL Simoa, median (IQR) | 128.98 (105.55, 135.29) | 119.53 (105.76, 133.52) | *p*=0.60 ^c^ |
| Aβ_42_ pg/mL Simoa, mean (sd) | 6.58 (2.25) | 7.70 (1.90) | ***p*=0.025 ^a^** |
| Aβ_42/40_ ratio Simoa, median (IQR) | 0.057 (0.044, 0.060) | 0.063 (0.059, 0.069) | ***p*=0.0003** ^c^ |
| p-tau-181 pg/mL, median (IQR) | 29.18 (22.35, 39.57) | 20.03 (15.75, 24.87) | ***p*=0.0001** ^c^ |
| GFAP pg/mL, median (IQR) | 71.97 (43.09, 131.03) | 79.17 (52.86, 110.00) | *p*=0.75 ^c^ |
| NfL pg/mL, median (IQR) | 40.96 (24.83, 65.93) | 17.86 (14.97, 21.53) | ***p*<0.0001** ^c^ |
| **MRI Markers of CAA** | | | |
| 0 – 1 CMBs Present | 5 (15.2%) | 32/32 (100.0%) | ***p*<0.0001^b^** |
| 2 – 4 CMBs Present | 6 (18.2%) | 0/32 (0.0%) |  |
| ≥ 5 CMBs Present | 22 (66.7%) | 0/32 (0.0%) |  |
| Any cSS | 22 (66.7%) | 0/32 (0.0%) | ***p*<0.0001^b^** |
| Focal cSS | 10 (30.3%) | 0/32 (0.0%) | ***p*=0.001^b^** |
| Disseminated cSS | 12 (36.4%) | 0/32 (0.0%) | ***p*<0.0001^b^** |
| High WMH | 27 (81.8%) | 9/32 (28.1%) | ***p*<0.0001^b^** |
| High CSO-PVS | 27 (81.8%) | 16/32 (50.0%) | ***p*=0.009^b^** |
| Median CAA SVD Score (IQR) | 4 (4, 5) | 1/32 (0, 1) | ***p*<0.0001 ^b^** |

**Abbreviations:** CAA, cerebral amyloid angiopathy; ICH, Intracerebral Hemorrhage; TFNEs, transient focal neurological episodes; Simoa, single molecule array; Aβ, amyloid beta; p-tau, phosphorylated tau; GFAP, glial fibrillary acidic protein; NfL, neurofilament light; CMBs, cortical microbleeds; cSS, cortical superficial siderosis, WMH, white matter hyperintensities; CSO-PVS high centrum semiovale perivascular space count. Superscripts indicate which statistical test was used: (a) Student’s t-test used when continuous variables were normally distributed (b) Fisher’s Exact Chi-Squared test (c) Mann-Whitney U Test when ordinal data compared or when distributions of continuous variables in either cohort were non-normally distributed.

**Supplemental Table 4:** Demographic, Vascular Risk Factor, Cognitive and Neuroimaging Data comparing those with insufficient (n=28) sample to those with sufficient sample (n=92)

| **Variable** | **Insufficient Sample** (n=28) | **Sufficient Sample** (n=92) | **p-value** |
| --- | --- | --- | --- |
| **Demographics** | | | |
| Age (years), mean(sd) | 69.43 (5.57) | 71.97 (7.13) | *p*=0.09^a^ |
| Sex, female | 11 (39.29%) | 50 (54.35%) | *p*=0.20^b^ |
| CAA Diagnosis | 8 (28.57%) | 45 (48.91%) | *p*=0.08 ^b^ |
| Years of Education, mean(sd) | 16.05 (3.36) | 15.17 (3.24) | *p*=0.21^a^ |
| **Vascular Risk Factors** | | | |
| Hypertension | 10/27 (37.0%) | 40 (43.5%) | *p*=0.66 ^b^ |
| Dyslipidemia | 8 (29.6%) | 39 (42.4%) | *p*=0.27 ^b^ |
| Diabetes | 3/27 (11.1%) | 6 (6.5%) | *p*=0.42 ^b^ |
| Smoking history (any) | 9/27 (33.3%) | 38/88 (43.2%) | *p*=0.50 ^b^ |
| **MRI Markers of CAA** | | | |
| 0 – 1 CMBs Present | 21/27 (77.8%) | 51/90 (56.7%) | ***p*=0.02 ^b^** |
| 2 – 4 CMBs Present | 4/27 (14.8%) | 9/90 (10.0%) |  |
| ≥ 5 CMBs Present | 2/27 (7.4%) | 30/90 (33.3%) |  |
| Any cSS | 3/27 (11.1%) | 28/90 (76.9%) | ***p*=0.047 ^b^** |
| Focal cSS | 2/27 (7.4%) | 12/90 (13.3%) | *p*=0.52 ^b^ |
| Disseminated cSS | 1/27 (3.7%) | 15/90 (16.7%) | *p*=0.11 ^b^ |
| High WMH | 11/27 (40.7%) | 49/90 (54.4%) | *p*=0.27 ^b^ |
| High CSO-PVS count | 16/27 (59.3%) | 57/90 (63.3%) | *p*=0.82 ^b^ |
| Median CAA SVD Score (IQR) | 1 (1, 2) n=27 | 2 (1, 4) n=90 | *p*=0.10 ^c^ |

Superscripts indicate which statistical test was used: (a) Student’s t-test used when continuous variables were normally distributed (b) Fisher’s Exact Chi-Squared test (c) Mann-Whitney U Test when ordinal data compared or when distributions of continuous variables in either cohort were non-normally distributed.

**Supplemental Table 5:** Comparison of demographic and vascular risk factors for n=11 that failed C2N quality control and n=81 who passed C2N quality control

| **Variable** | **Passed C2N Quality Control** (n=81) | **Failed C2N Quality Control** (n=11) | **p-value** |
| --- | --- | --- | --- |
| Age | 72.01 (7.18) | 71.69 (7.04) | *p*=0.89 ^a^ |
| Sex, female | 45 (55.6%) | 5 (45.5%) | *p*=0.54 ^b^ |
| CAA Diagnosis | 41 (50.6%) | 4 (36.4%) | *p*=0.52 ^b^ |
| Years of Education | 15.07 (3.32) | 15.91 (2.66) | *p*=0.42 ^a^ |
| Hypertension | 33 (40.7%) | 7 (63.6%) | *p*=0.20 ^b^ |
| Dyslipidemia | 31 (38.3%) | 8 (72.7%) | ***p*=0.048** ^b^ |
| Diabetes | 4 (4.9%) | 2 (18.2%) | *p*=0.15 ^b^ |
| Smoking history (any) | 34/77 (44.2%) | 4 (36.4%) | *p*=0.75 ^b^ |

Superscripts indicate which statistical test was used: (a) student’s t-test used when continuous variables were normally distributed (b) Fisher’s Exact Chi-Squared test (c) Mann-Whitney U Test when ordinal data compared or when distributions of continuous variables in either cohort were non-normally distributed

**Supplementary Table 6:** Linear regression models with age and sex adjusted standardized beta-coefficients for plasma biomarker values in CAA vs healthy controls

| **Plasma Biomarker** | **Model** | **n** | **β-CAA** | **S.E** | **95% CI:** | **p-value** |
| --- | --- | --- | --- | --- | --- | --- |
| **Z-score Aβ_42/40_ IPMS** | 1^a^ | 81 | -0.67 | 0.21 | -1.09, -0.25 | *p*=**0.002** |
|  | 1^b^ | 81 | -0.60 | 0.22 | -1.04, -0.15 | *p*=**0.009** |
|  | 1^c^ | 81 | -0.39 | 0.22 | -0.83, 0.04 | *p*=0.075 |
| **Z-score Aβ_42/40_ Simoa** | 2^a^ | 75 | -0.77 | 0.21 | -1.20, -0.34 | *p*=**0.001** |
|  | 2^b^ | 75 | -0.71 | 0.22 | -1.15, -0.27 | *p*=**0.002** |
|  | 2^c^ | 75 | -0.69 | 0.23 | -1.15, -0.23 | *p*=**0.004** |
| **Z-score log NfL pg/mL*** | 3^a^ | 75 | 1.06 | 0.20 | 0.67, 1.45 | *p***<0.0001** |
|  | 3^b^ | 75 | 0.86 | 0.19 | 0.49, 1.24 | *p***<0.0001** |
|  | 3^c^ | 75 | 0.93 | 0.19 | 0.54, 1.31 | *p***<0.0001** |
| **Z-score log p-tau-181 pg/mL*** | 4^a^ | 75 | 1.02 | 0.20 | 0.62, 1.42 | *p***<0.0001** |
|  | 4^b^ | 75 | 0.92 | 0.20 | 0.51, 1.33 | *p***<0.0001** |
|  | 4^c^ | 75 | 0.75 | 0.20 | 0.349, 1.14 | *p***<0.0001** |
| **Z-score log GFAP pg/mL*** | 5^a^ | 75 | 0.08 | 0.23 | -0.38, 0.54 | *p*=0.735 |
|  | 5^b^ | 75 | -0.19 | 0.24 | -0.50, 0.46 | *p*=0.938 |
|  | 5^c^ | 75 | 0.11 | 0.24 | -0.37, 0.60 | *p*=0.641 |

a. unadjusted model b. model solely adjusted for age c. model adjusted for age and sex

* log transformation of the plasma biomarker concentration to improve normality of residuals and/or homoscedasticity in regression models, which was subsequently transformed to a Z score.

**Supplemental Table 7:** Proportion of Healthy Controls and those with CAA falling into age specific plasma concentration percentiles derived from n=900 in Cooper et al.^1^ from the ageing Canadian population for ages 3 – 79 years. Here the <5^th^ percentile was defined as anything less than the lower limit of the 95% CI of the predicted 5^th^ percentile for each age, while >95th percentile was defined as anything greater than the upper limit of the 95% CI of the predicted 95^th^ percentile for each age.

| **Biomarker** | **Group** | **5^th^ – 95^th^ percentile**  **Count(%)** | **<5^th^ percentile**  **Count(%)** | **>95^th^ percentile**  **Count(%)** | **Fisher’s Exact Chi-Squared p-value** |
| --- | --- | --- | --- | --- | --- |
| Aβ_42/40_ | healthy controls | 38/39 (97.44%) | 1/39 (2.56%) | 0/39 (0.00%) | *p*=0.014 |
|  | CAA | 28/36 (77.77%) | 7/36 (19.44%) | 1/36 (2.78%) |  |
| NfL | healthy controls | 35/39 (89.74%) | 1/39 (2.56%) | 3/39 (7.69%) | *p*<0.0001 |
|  | CAA | 19/36 (52.78%) | 0/36 (0.00%) | 17/36 (47.22%) |  |
| p-tau-181 | healthy controls | 33/39 (84.62%) | 5/39 (12.82%) | 1/39 (2.56%) | *p*=0.076 |
|  | CAA | 28/36 (77.78%) | 2/36 (5.56%) | 6/36 (16.67%) |  |
| GFAP | healthy controls | 33/39 (84.62%) | 5/39 (12.82%) | 1/39 (2.56%) | *p*=0.029 |
|  | CAA | 23/36 (69.44%) | 13/36 (36.11%) | 0/36 (0.00%) |  |

**Supplemental Table 8:** Number and Proportions of Healthy Controls and those with CAA categorized as having aberrantly low (<5^th^ percentile) Aβ_42/40_ or aberrantly high (>95^th^ percentile) elevations in p-tau-181, NfL, or GFAP (classified according to population reference intervals from Cooper et al.^1^)

| **Biomarker** | **Group** | **Test Negative**  $\geq$5%tile | **Test Positive**  <5%tile | **OR** | **95% CI** | **p-value** |
| --- | --- | --- | --- | --- | --- | --- |
| Aβ_42/40_ | healthy controls | 38/39 (97.44%) | 1/39 (2.56%) | 9.17 | 1.07, 78.77 | *p*=0.018 |
|  | CAA | 29/36 (80.56%) | 7/36 (19.44%) |  |  |  |
| **Biomarker** | **Group** | **Test Negative**  $\leq$95%tile | **Test Positive**  >95%tile | **OR** | **95% CI** | **p-value** |
| NfL | healthy controls | 36/39 (92.31%) | 3/39 (7.69%) | 10.74 | 2.79, 41.31 | *p*=0.0001 |
|  | CAA | 19/36 (52.78%) | 17/36 (47.22%) |  |  |  |
| p-tau-181 | healthy controls | 38/39 (97.44%) | 1/39 (2.56%) | 7.6 | 0.87, 66.59 | *p*=0.036 |
|  | CAA | 30/36 (83.33%) | 6/36 (16.67%) |  |  |  |
| GFAP | healthy controls | 37/39 (94.87%) | 2/39 (5.13%) | 0.0 | - | *p*=0.17 |
|  | CAA | 36/36 (100.00%) | 0/36 (0.00%) |  |  |  |

**Supplemental Table 9:** Number and Proportions of Healthy controls and those with CAA categorized with GFAP <5^th^ percentile (classified according to population reference intervals from Cooper et al.^1^)

| **Biomarker** | **Group** | $\boldsymbol{\geq}$**5^th^ percentile** | **<5^th^ percentile** | **OR** | **95% CI** | **p-value** |
| --- | --- | --- | --- | --- | --- | --- |
| GFAP | healthy controls | 34/39 (84.62%) | 5/39 (12.8%) | 3.84 | 1.21, 12.25 | *p*=0.018 |
|  | CAA | 23/36 (63.89%) | 13/36 (36.11%) |  |  |  |

**Supplemental Table 10:**  Logistic Regression model beta-values (log-odds) of plasma-biomarkers in pg/mL and Youden’s Index defined univariate cut-points

| **Model** | **Model Terms** | **β-coefficient** | **β 95% CI** | **Cut point** |
| --- | --- | --- | --- | --- |
| Model 1 | Aβ_42/40_ IPMS | -81.22 | -136.73, -25.71 | 0.095 |
|  | *Constant Term* | 7.65 | 2.43, 12.87 |  |
| Model 2 | Aβ_42/40_ Simoa | -79.01 | -28.38, -129.64 | 0.059 |
|  | *Constant Term* | 4.57 | 7.60, 1.54 |  |
| Model 3 | NfL Simoa (pg/mL) | 0.063 | 0.028, 0.097 | 23.22 pg/mL |
|  | *Constant Term* | -1.98 | -3.05, -0.92 |  |
| Model 4 | p-tau-181 Simoa (pg/mL) | 0.129 | 0.060, 0.197 | 32.24 pg/mL |
|  | *Constant Term* | -3.38 | -5.14, -1.62 |  |
| Model 5 | Aβ_42/40_ IPMS | -11.62 | -86.93, 63.69 | 0.392 |
|  | NfL (pg/mL) | 0.087 | 0.028, 0.146 |  |
|  | p-tau-181 (pg/mL) | 0.067 | -0.019, 0.152 |  |
|  | *Constant Term* | -3.04 | -11.17, 5.09 |  |
| Model 6 | Aβ_42/40_ Simoa | -86.33 | -142.24, -30.43 | 0.359 |
|  | NfL (pg/mL) | 0.049 | 0.009, 0.089 |  |
|  | p-tau-181 (pg/mL) | 0.094 | 0.022, 0.165 |  |
|  | *Constant Term* | 1.11 | -2.19, 4.41 |  |

**Supplemental Table 11: Subgroup Analysis: excluding those with CAA presenting with cognitive impairment**

| **Variable** | **CAA** (n=35) | **Healthy controls** (n=47) | **p-value** |
| --- | --- | --- | --- |
| **Demographics** | | | |
| Age (years), mean (sd) | 74.2 (7.78) | 69.80 (6.43) | ***p*=0.006^a^** |
| Sex, female | 15 (42.9%) | 32 (68.1%) | ***p*=0.03^b^** |
| Years of Education, median (IQR) | 15 (12, 17) | 16 (13.5,17) | *p*=0.08^c^ |
| **Plasma Biomarkers** | | | |
| Aβ_40_ (IPMS) pg/mL, median (IQR) | 446.31 (409.85, 484.03) n=33 | 444.63 (395.85, 472.38) n=40 | *p*=0.78^c^ |
| Aβ_42_ (IPMS) pg/mL, median (IQR) | 39.82 (35.57, 42.54) n=33 | 43.40 (38.45, 46.79) n=40 | *p*=0.09^c^ |
| Aβ_42/40_ (IPMS) ratio, mean (sd) | 0.091 (0.009) n=33 | 0.097 (0.009) n=40 | ***p*=0.009 ^a^** |
| Aβ_40_ (Simoa) pg/mL, median (IQR) | 122.39 (104.50, 132.40) n=29 | 119.53 (105.30, 134.06) n=39 | *p*=0.97 ^c^ |
| Aβ_42_ (Simoa) pg/mL, mean (sd) | 6.29 (2.23) n=29 | 7.76 (1.89) n=39 | ***p*=0.005 ^a^** |
| Aβ_42/40_ (Simoa) ratio, median (IQR) | 0.057 (0.044, 0.060) n=29 | 0.064 (0.060, 0.069) n=39 | ***p*<0.0001** ^c^ |
| p-tau-181 pg/mL, median (IQR) | 29.16 (21.37, 39.57) n=29 | 20.03 (15.63, 24.87) n=39 | **p=0.0002** ^c^ |
| GFAP pg/mL, median (IQR) | 68.30 (40.34, 130.67) n=29 | 79.17 (54.47, 108.45) n=39 | *p*=0.53^c^ |
| NfL pg/mL, median (IQR) | 38.87 (23.26, 60.02) n=29 | 17.86 (14.97, 23.19) n=39 | ***p*<0.0001** ^c^ |

**Abbreviations:** CAA, cerebral amyloid angiopathy; IPMS, immunoprecipitation mass spectrometry; Simoa, single molecule array; Aβ, amyloid beta; p-tau, phosphorylated tau; GFAP, glial fibrillary acidic protein; NfL, neurofilament light chain. Superscripts indicate which statistical test was used: (a) Student’s t-test used when continuous variables were normally distributed (b) Fisher’s Exact Chi-Squared test (c) Mann-Whitney U Test when ordinal data compared or when distributions of continuous variables in either cohort were non-normally distributed.

#

# **SUPPLEMENTARY FIGURES:**

**Supplementary Figure 1:** Patient flow diagram of consented participants in FAVR-II included in the IPMS beta-amyloid plasma biomarker analysis from C_2_N. Abbreviations: Healthy Controls (HC); Cerebral Amyloid angiopathy (CAA)

**Supplementary Figure 2:** Patient flow diagram of consented participants included in the Simoa beta-amyloid (Quanterix Ltd), p-tau-181, NfL and GFAP plasma biomarker analysis. Abbreviations: Healthy Controls (HC); Cerebral Amyloid angiopathy (CAA)

**REFERENCES**

1. Cooper JG, Stukas S, Ghodsi M, et al. Age specific reference intervals for plasma biomarkers of neurodegeneration and neurotrauma in a Canadian population. Clin Biochem 2023;121-122:110680.

2. Quanterix. Simoa p-tau 181 Advantage V2.1 Assay (reference TECH-0153). 2022. Available at: <https://portal.quanterix.com/folder-viewer/Assay%20Technical%20Notes/Bead%20Assays>.
